# Imatinib mesylate inhibits STAT5 phosphorylation in response to IL-7 and promotes T cell lymphopenia in chronic myelogenous leukemia patients

**DOI:** 10.1038/bcj.2017.29

**Published:** 2017-04-07

**Authors:** S Thiant, M M Moutuou, P Laflamme, R Sidi Boumedine, D M Leboeuf, L Busque, J Roy, M Guimond

**Affiliations:** 1 Division d'Hématologie-Oncologie, Centre de Recherche de l'Hôpital Maisonneuve-Rosemont, Montréal, Québec, Canada; 2 Départment de Microbiologie, Infectiologie et Immunologie, Université de Montréal, Montréal, Québec, Canada; 3 Départment de Médecine, Université de Montréal, Montréal, Québec, Canada

## Abstract

Imatinib mesylate (IM) therapy has been shown to induce lower T cell counts in chronic myelogenous leukemia (CML) patients and an interference of IM with T cell receptor (TCR) signaling has been invoked to explain this observation. However, IL-7 and TCR signaling are both essential for lymphocyte survival. This study was undertaken to determine whether IM interferes with IL-7 or TCR signaling to explain lower T cell counts in patients. At diagnosis, CML patients have typically lower CD4^+^ counts in their blood, yet CD8^+^ counts are normal or even increased in some. Following the initiation of IM treatment, CD4^+^ counts were further diminished and CD8^+^ T lymphocytes were dramatically decreased. *In vitro* studies confirmed IM interference with TCR signaling through the inhibition of ERK phosphorylation and we showed a similar effect on IL-7 signaling and STAT5 phosphorylation (STAT5-p). Importantly however, using an *in vivo* mouse model, we demonstrated that IM impaired T cell survival through the inhibition of IL-7 and STAT5-p but not TCR signaling which remained unaffected during IM therapy. Thus, off-target inhibitory effects of IM on IL-7 and STAT5-p explain how T cell lymphopenia occurs in patients treated with IM.

## Key points

- Imatinib disrupts T cell homeostasis through the inhibition of IL-7 and STAT5 phosphorylation.
- Imatinib attenuates cytokine signaling in different clinical settings of immune dysfunctions.

## Introduction

Imatinib mesylate (IM) is currently the drug of choice for first line therapy in patients with Philadelphia chromosome-positive chronic myelogenous leukemia (CML). Despite the relatively high specificity of IM treatment towards the BCR-ABL fusion protein, off-target multikinase inhibitory effects occur and can interfere with normal hematopoiesis.^1, 2^ For instance, non-specific inhibition of Flt3L has been associated with disruption of dendritic cell (DC) homeostasis and functions in both mice and humans.^3^ In addition, studies have reported an interference of IM with T cell counts and activation.^4^ T lymphocytes require T cell receptor (TCR) stimulation by MHC-I or MHC-II and IL-7 signaling in order to survive and persist in the periphery. While TCR signaling induces the phosphorylation and activation of AKT by the lipid kinase phosphatidylinositol 3-kinase (PI3K), IL-7 signaling induces the phosphorylation of STAT5 (STAT5-p) by Jak1-3 protein kinases; these pathways constitute potential targets for IM.^5, 6^ Despite ample evidence that IM can inhibit TCR signaling *in vitro*, the precise mechanism of action of IM on T cell homeostasis has remained inconclusive *in vivo*. The present studies were undertaken to precise the impact of IM on TCR or IL-7 inhibition in order to explain T cell lymphopenia in CML patients.

## Materials and methods

### Clinical samples

Healthy donors (*n*=25), CML patients at diagnosis (*n*=22) and patients treated with IM (*n*=10) were recruited. Patients treated with IM received between 200 and 600 mg/day. The median time of IM treatment was 2.9 years (range: 0.5–10.9) and the median time of remission post-IM was 1.1 years (range: 0.3–3). Blood samples were obtained under protocols approved by the HMR Ethics Committee, and written informed consent was obtained from all patients and healthy donors.

### Flow cytometry analyses

The percentage and absolute counts of naive CD4^+^ or CD8^+^ T lymphocytes; (CD3^+^CD45RA^+^CCR7^+^), central memory; (CD3^+^CD45RA^+^CCR7^neg^) and effector memory (T_EM_); (CD3^+^CD45RA^neg^CCR7^neg^) were determined by flow cytometry. FITC-CD3, Pacific Blue-CD4, APCcy7-CD8, APC-CCR7, PEcy7-CD45RA were used to evaluate naive and memory T cells (BD Bioscience, San Diego, CA, USA). PE-CD56 was used to evaluate NK cells (BD Biosciences). Non-specific binding was determined using isotypic controls. Flow cytometry acquisition was performed on LSRII and analysis with Flowjo software (Treestar, Ashland, OR, USA).^7^ The percentages and absolute T-cell counts were calculated based on lymphocytes and monocytes gating.

### IL-7 and TCR signaling

PBMCs from normal subjects were cultured at 2 × 10^6^ cells/ml in RPMI+10% FCS and incubated with IM (3 μmol/ml) at 37 °C for 24 h.^8^ For STAT5 phosphorylation, cells were washed and incubated in serum-free medium for 1 h before stimulation with rhIL-7 (Cytheris) for 30 min at 37 °C. After fixation and permeabilization in Perm-Buffer III (BD Biosciences),^9^ cells were stained for STAT-5p (PE-STAT5, clone pY694, BD Biosciences) and cell surface receptors. For ERK phosphorylation (PE-ERK1-2, T202/pY204, BD Biosciences) following anti-CD3 stimulation (OKT3; BioXCell, West Lebanon, NH, USA), cells were serum starved for 1 h, incubated with 1 mg/ml of anti-CD3 for 15 min and then cross-linked with anti-mouse immunoglobulin-G (Sigma, Oakville, ON, Canada) at 5 mg/ml for 15 min. Intracellular staining was performed as described above.

### Mouse studies

All experiments were approved by the Animal Ethic Committee of the HMR. For adoptive transfer of T cells into Rag^−/−^ mice (CD45.2^+^), 1 × 10^6^ of enriched lymph node T cells (CD45.1^+^) were labeled with cell trace violet (Invitrogen, Burlington, ON, Canada) and adoptively transferred through the tail vein.^7^ Mice were treated by gavage with IM 100 mg/kg twice a day for 7 days. Cell trace violet content was analyzed in CD45.1^+^ T cells by flow cytometry.

### Statistical analysis

Prism 5.0 (GraphPad, La Jolla, CA, USA) was used for statistical analysis. The Mann Whitney U test was used to compare paired data, whereas the Kruskal–Wallis test followed by Dunn's post-test was used to compare three or more groups.

## Results and discussion

**Figure 1:**
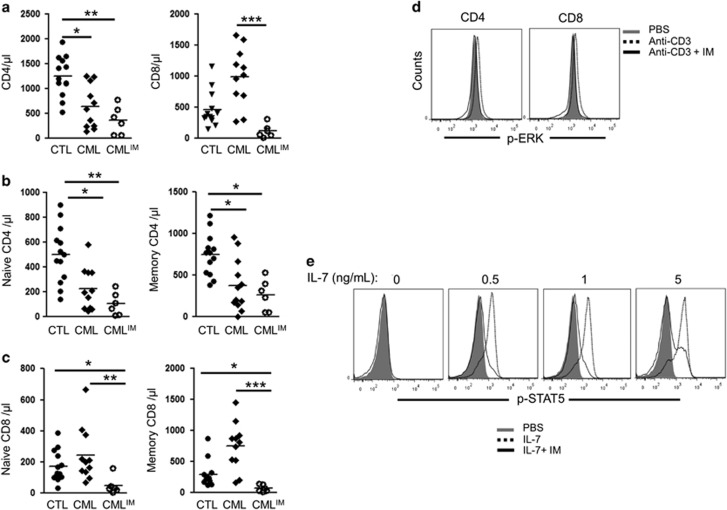
(**a**) Graphical summary of the absolute number (cells/μl) of CD4^+^ T cells and CD8^+^ T cells enumerated in the blood of age match controls (*n*=12), CML at diagnosis (*n*=11) and CML patients treated by TKI (*n*=6). (**a**–**c**) Absolute counts of naive (CCR7^+^CD45^+^ cells) and memory (CCR7^+^CD45RA^neg^, CCR7^neg^CD45RA^neg^ and CCR7^neg^CD45RA^+^) CD4^+^ and CD8^+^ T cells. Differences between groups were assessed by Kruskal-Wallis *U* test (Dunns post-test). *P<0.05; ** *P*<0.01; ***P<0.001. (**d**) Evaluation of ERK phosphorylation in CD4^+^ and CD8^+^ T cells incubated with 3 μM IM and then exposed to anti-CD3 stimulation. Results are representative of two independent experiments. (**e**) Evaluation of STAT5 phosphorylation in T cells incubated overnight with IM 3 μM and then exposed to varying concentrations of rhIL-7 (ng/ml). Results are representative of three independent experiments. Differences between groups were assessed by Kruskal-Wallis U test (Dunns post-test). *P<0.05; **P<0.01; ***P<0.001.

Lymphopenia has been reported in CML patients under IM therapy,^10^ and T cells were evaluated before and after IM treatment. At diagnosis, CML patients have lower naive and memory CD4^+^ T cells, while CD8^+^ counts were not diminished. Following IM treatment, CD4^+^ counts remained diminished and naive and memory CD8^+^ T cells were much lower (Figures 1a–c). We confirmed an effect of IM with TCR signaling^4^ by showing the inhibition of ERK-1/2 phosphorylation in response to suboptimal TCR simulation in CD4^+^ and CD8^+^ T cells exposed to 3 μM of IM (Figure 1d). As IL-7 is essential for naive and memory T cell survival,^9, 11^ we evaluated IM effect on STAT5-p in response to IL-7 stimulation in T cells. While 0.5 and 1 ng/ml of IL-7 are sufficient for inducing STAT5-p in T cells, much higher concentrations of IL-7 are required when these cells are exposed to 3 μM of IM (Figure 1e). Thus, in addition to TCR stimulation, our *in vitro* studies confirm that IM can interfere with IL-7 signaling and STAT5-p in T cells.

**Figure 2:**
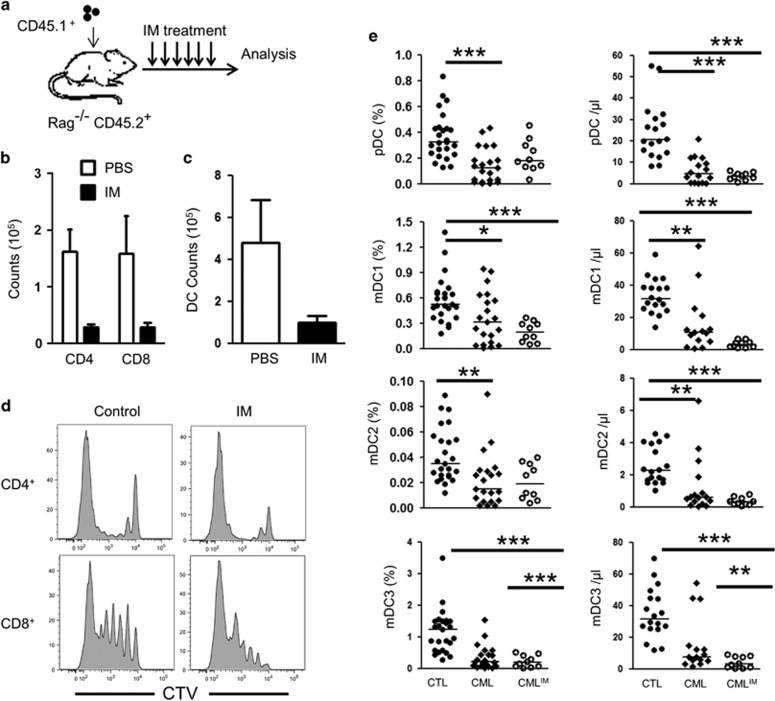
(**a**) Schematic representation of the *in vivo* mouse model to evaluate IM effect on T cells. (**b**) Absolute numbers of congenic CD4^+^ and CD8^+^ T cells recovered in IM treated mice. (**c**) Absolute numbers of CD11c^+^ DCs after 7 days of IM treatment. (**d**) CD4^+^ and CD8^+^ T cell proliferation 7 days after transfer into lymphopenic recipients treated or not with IM. Data are representative of two independent experiments, four mice per group. (**e**) Graphical summary of the median percentage (left) and absolute counts (right) of pDC (HLADR^+^CD14^neg^CD303^+^CD123^+^), mDC1 (HLADR^+^CD14^neg^CD11c^hi^CD1c^+^), mDC2 (HLADR^+^CD14^neg^CD11c^+^CD141c^hi^), mDC3 (HLADR^+^CD14^neg^CD11c^hi^CD16^+^) in the blood of healthy subjects (*n*=25), CML at diagnosis (*n*=22), and CML patients treated by IM (*n*=10).

While lower systemic IL-7 concentrations induce survival through Stat5-p and BCL-2 synthesis,^12, 13^ higher concentrations of IL-7 signal through PI3K and synergize with TCR signaling to induce homeostatic proliferation of T cells.^14, 15^ We therefore used lymphopenic Rag^−/−^ mice to evaluate the *in vivo* effect of IM on survival and homeostatic proliferation of adoptively transferred T lymphocytes (Figure 2a). After 7 days of IM treatment, lymphocyte counts were much lower, indicating a potential defect in survival and/or homeostatic proliferation of transferred T cells (Figure 2b). Importantly, IM treatment did not reduce homeostatic proliferation of T cells, thus confirming that TCR signaling remains functional during IM treatment and supporting a model wherein the loss of T cells is predominantly mediated through the inhibition of IL-7 signaling and STAT5-p (Figure 2d). Previous studies have invoked a potential role for TCR inhibition by IM to explain diminished delayed type hypersensitivity in mice.^16^ However, we showed herein that IM induces DC depletion in humans and mice and this could contribute to limit delayed type hypersensitivity development (Figures 2c and e).^17, 18^ Furthermore, IL-7 can act as an adjuvant to facilitate T cell activation and the inhibition of IL-7 signaling could probably impair delayed type hypersensitivity response.^19, 20^ Finally, while the inhibition of TCR signaling by IM could perhaps explain lower naive CD4^+^, CD8^+^ and memory CD4^+^ T cell counts, it does not explain lower memory CD8^+^ counts, as these cells do not require TCR stimulation for their peripheral maintenance.^21^ Thus, despite conclusive *in vitro* evidences showing an interference of IM with TCR signaling,^4, 16, 22^ our *in vivo* data are more consistent with an effect of IM on STAT5 to explain lower T cell counts. STAT5 is required for the signaling of other cytokines and it is possible that the effect of IM on T cells is not entirely restricted to IL-7 signaling.^23^ Additional studies are needed in order to understand the full spectrum of cytokines signaling inhibited by IM.

In conclusion, this study has several potential clinical implications. First, lymphopenia occurs in most CML patients following IM treatment. The use of IL-7 therapy could perhaps improve T cell counts in patients at higher risk of infections such as those receiving IM for persistent disease after allogeneic stem cell transplant.^24^ Second, although promising results were initially obtained for the treatment of steroid refractory chronic graft-versus-host disease, <50% of patients respond to IM therapy and its use has not been widely accepted in the transplant community.^25, 26^ IL-7 can facilitate T cell activation but once activated, IL-7 receptor is down-modulated; this could limit the IM effect on T cells involved in chronic graft-versus-host disease and explain the disappointing results observed clinically. Third, in acute lymphoblastic leukemia, IL-7 can contribute to the survival of acute lymphoblastic leukemia cells and it is not excluded that part of IM effectiveness in Philadelphia-positive acute lymphoblastic leukemia cells may be related to the inhibition of IL-7 signaling and stat5p.^27, 28^ However, the benefit of IM in Philadelphia-positive acute lymphoblastic leukemia patients who do not undergo allogeneic transplant is often of a short duration, and rises in systemic IL-7 that typically occurs during lymphopenia may reduce the effectiveness of IM at blocking stat5p (Figure 1e).^29^ In contrast, decrease in systemic IL-7 occurs following allogeneic stem cell transplantation despite profound lymphopenia, and IM treatment can still successfully prevent relapse in most patients.^7, 30, 31^ Whether IL-7 can induce IM resistance after allogeneic transplant remains unknown. Nonetheless, the finding that IL-7-stat5p can be inhibited by IM provides a novel perspective on IM treatment that should be further studied in future clinical trials.
