# Correction: Imatinib mesylate inhibits STAT5 phosphorylation in response to IL-7 and promotes T cell lymphopenia in chronic myelogenous leukemia patients

**DOI:** 10.1038/s41408-020-00374-3

**Published:** 2020-11-04

**Authors:** S. Thiant, M. M. Moutuou, P. Laflamme, R. Sidi Boumedine, D. M. Leboeuf, L. Busque, J. Roy, M. Guimond

**Affiliations:** 1 Division d’Hématologie-Oncologie, Centre de Recherche de l’Hôpital Maisonneuve-Rosemont, Montréal, Québec Canada; 2 Départment de Microbiologie, Infectiologie et Immunologie, Université de Montréal, Montréal, Québec Canada; 3 Départment de Médecine, Université de Montréal, Montréal, Québec Canada; 4 Present Address: Skolkovo Institute of Science and Technology, Moscow, Russia

Correction to: *Blood Cancer Journal*

10.1038/bcj.2017.29 published online 7 April 2017

Following the publication of this article the authors noted that Grant numbers were omitted in the Acknowledgements section. This work was supported by grants from the Cancer Research Society of Canada (grants no. 16255 to M.G.) and from the Fond de Recherche Santé Québec (grant no 20219 to J.R.) and in part by the Foundation de l’Hôpital Maisonneuve-Rosemont, Montreal, QC, CAN. The authors wish to apologize for any inconvenience caused.

